# Molecular insights into Sertoli cell function: how do metabolic disorders in childhood and adolescence affect spermatogonial fate?

**DOI:** 10.1038/s41467-024-49765-1

**Published:** 2024-07-03

**Authors:** Rossella Cannarella, Roberto Curto, Rosita A. Condorelli, Scott D. Lundy, Sandro La Vignera, Aldo E. Calogero

**Affiliations:** 1 Department of Clinical and Experimental Medicine, University of Catania, Catania, Italy; 2 Glickman Urological & Kidney Institute, Cleveland Clinic Foundation, Cleveland, OH USA

**Keywords:** Gonadal disorders, Cell signalling

## Abstract

Male infertility is a major public health concern globally with unknown etiology in approximately half of cases. The decline in total sperm count over the past four decades and the parallel increase in childhood obesity may suggest an association between these two conditions. Here, we review the molecular mechanisms through which obesity during childhood and adolescence may impair future testicular function. Several mechanisms occurring in obesity can interfere with the delicate metabolic processes taking place at the testicular level during childhood and adolescence, providing the molecular substrate to hypothesize a causal relationship between childhood obesity and the risk of low sperm counts in adulthood.

## Abstract (web summary)

Pediatric obesity is increasing worldwide and its contribution to the decline is sperm count has been questioned. By comprehensively reviewing the literature, the authors herein report molecular mechanisms that may suggest the causality of this association.

## Introduction

According to the World Health Organization (WHO), the worldwide prevalence of overweight and obesity has nearly tripled since 1975^1^. The prevalence of overweight and obesity has also reached alarming numbers in childhood. Indeed, more than 340 million children and adolescents aged between 5 and 19 were overweight or obese in 2016^1^.

When body fat reaches pathological levels, adipocytes induce the onset of chronic low-grade systemic inflammation by releasing proinflammatory cytokines and free fatty acids. These molecules antagonize insulin-mediated metabolic processes and cause insulin resistance. Overweight and obesity, therefore, represent the main risk factors for insulin resistance, which in turn causes a progressive increase in its secretion by pancreatic β-cells, leading to hyperinsulinemia^2^.

Growing evidence suggests that being overweight or obese negatively affects male reproductive function^3^. In fact, several studies have demonstrated the negative impact of excess weight on sperm parameters^4–6^.

Epidemiologically, the decline in sperm counts recorded over the last 40 years^7^ and the concomitant increase in the prevalence of pediatric obesity support the theoretical existence of a causal relationship between the two phenomena. However, the mechanisms underlying the relationship between obesity in childhood and adolescence and the risk of developing male infertility in adulthood are not yet fully understood and require further studies.

Here, we provide an overview of the effects on follicle-stimulating hormone (FSH), insulin-like growth factor (IGF) system, androgen, and estrogen signaling pathways on Sertoli cells (SC) function and the “niche” microenvironment, and an overview of molecular interference associated with obesity and insulin resistance all within the main signaling pathway involved in the proliferation and maturation of SCs and germ cells (GCs), which could be the basis for establishing a causal relationship between childhood obesity and the risk of reduced sperm count in adulthood. Finally, evidence on the interplay between glucose metabolism, obesity-related inflammation, adipocytokines, gut hormones, and SC function, and the spermatogenetic niche is also discussed.

## Molecular signaling pathway

### Follicle-stimulating hormone

FSH is a glycoprotein made up of two subunits: α and β. The α subunit is shared with other pituitary hormones, while the β subunit provides specificity for FSH receptor (FSHR)^8^.

In the “niche” microenvironment, FSHR is expressed only on the cell membrane of SCs^9^. FSH and its receptor play a crucial role in regulating the proliferation, differentiation, and apoptosis of SCs, as well as promoting the maintenance, differentiation, and survival of the spermatogonial pool. These processes occur in specific stages during the development of SCs. Consequently, the response of SCs to FSH depends on their maturation status^10^.

FSH regulates SC proliferation during fetal and early postnatal life, thus determining the final number of SCs. During puberty, however, the SCs lose the ability to proliferate^10^, and FSH instead promotes the maturation of these cells.

#### Follicle-stimulating hormone regulates Sertoli cell proliferation, differentiation, and apoptosis

One of the best-studied effects of FSH on SC function is the regulation of their proliferation^10^. During early postnatal life, SC proliferation is triggered by activation of the cyclic adenosine monophosphate (cAMP)/protein kinase A (PKA) pathway, the extracellular regulated kinase (ERK)/mitogen-activated protein kinase (MAPK) pathway, and the phosphoinositide 3-kinase (PI3K)/ protein kinase B (Akt)/mammalian target of rapamycin complex 1 (mTORC1) pathway. Experimental evidence has demonstrated that incubation with dibutyryl-cAMP (dpcAMP) increases [^3^H]-thymidine incorporation in immature SCs^11^.

**Fig. 1:**
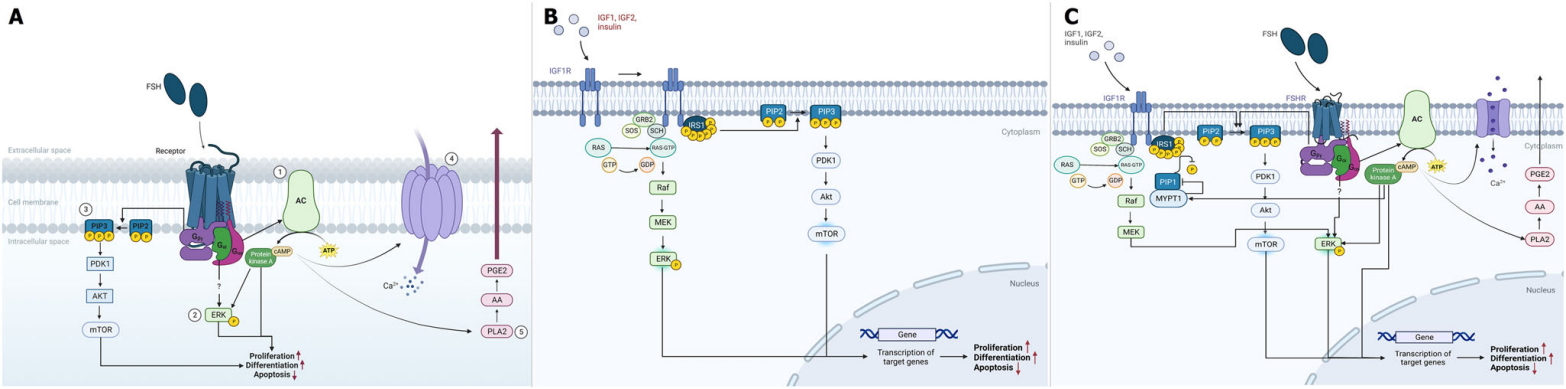
FSH and IGF1 signaling pathways. FSH signaling pathways (**A**). FSH binds FSHR on the cytoplasmic membrane of Sertoli cells. (1) FSHR couples to the Gɑs subunit to activate AC resulting in the activation of the cAMP/PKA pathway. (2) FSHR activates the ERK/MAPK pathway by coupling to the Gɑi and Gɑs subunits. (3) Coupling of FSHR to Gβγ activates the PI3K/AKT/mTOR signaling pathway. Furthermore, (4) the calcium pathway and 5) the PLA2 pathway are activated in a cAMP-dependent manner. IGF1R signaling pathway (**B**). PP1 is the hub link between the FSHR and IGF1R signaling pathway (**C**). The binding of FSH to its receptor activates AC through Gɑs. AC converts ATP to cAMP, which in turn activates PKA. PKA phosphorylates MYPT1 that results in an activation of PP1. Subsequently, the latter dephosphorylates inhibitory IRS1 *Ser/Thr* residues. Finally, IGF1, IGF2, or insulin, interacting with IGF1R, promote the phosphorylation of IRS1 which activates the PI3K/AKT/mTOR signaling pathway. AA arachidonic acid, AC Adenylate cyclase, AKT protein kinase B, ATP adenosine triphosphate, cAMP cyclic adenosine monophosphate, ERK extracellular signal-regulated kinase, FSH follicle-stimulating hormone, FSHR follicle-stimulating hormone receptor, Grb2 growth factor receptor-bound protein 2, IGF1 insulin-like growth factor 1, IGF2 insulin-like growth factor 2, IRS1 insulin receptor substrate 1, MAPK mitogen-activated protein kinase, Mek mitogen-activated protein kinase kinase, mTOR mammalian target of rapamycin, MYPT1 myosin phosphatase target subunit 1, PDK1 3-phosphoinositide-dependent kinase 1, PGE2 Prostaglandin E2, PIP2 phosphatidylinositol 4,5-bisphosphate, PIP3 phosphatidylinositol 3,4,5-trisphosphate, PI3K phosphoinositide 3-kinase, PKA cAMP-dependent protein kinase, PLA2 phospholipase A2, PP1 protein phosphatase 1.

FSH binds to its G protein-coupled receptor, which stimulates the cAMP/PKA pathway resulting in the phosphorylation of cyclic AMP response element binding protein (CREB), which in turn interacts with cAMP response element (CRE) in the promoter region of target genes regulating their gene expression^12^ (Fig. 1, panel A).

FSHR, by coupling Gα_s_ subunits, activates the ERK/MAPK pathway in a cAMP/PKA-dependent manner^13^. Furthermore, FSHR can activate the same pathway by coupling Gα_i_ subunits in a Src proto-oncogene non-receptor tyrosine kinase (Src)-dependent manner^13^. Once ERK is activated, it phosphorylates CREB, which in turn interacts with CRE, promoting gene expression (Fig. 1, panel A).

The most important signaling pathway during the proliferation phase of SCs is the PI3K/Akt/mTORC1^14^ (Fig. 1, panel A). The latter promotes the expression of specific genes involved in DNA replication and cell cycle progression, such as *cell-derived Myc* (*c-Myc*) and *type D1 cyclin* (*Cyclin-D1*).

The differentiation phase begins after the cessation of SC mitotic activity. This phase occurs during puberty, when SCs begin to express AR and the intratubular concentration of T increases, in a LH-dependent manner^15^. During this process, SCs form the blood-testis barrier (BTB), separate the SSCs and differentiated ones, and release nutrients for GCs. Unlike the proliferative phase, FSH mainly regulates the maturation process through the cAMP/PKA signaling pathway.

Regarding the formation of the BTB, FSH promotes the expression of HIF2, which up-regulates the expression of tight junction proteins ZO-1, ZO-2, Occludin^16^, and the expression of claudin-11, N-cadherin, and α6β1-integrin^17,18^ which are components of the BTB, through an unknown pathway. Furthermore, *tissue plasminogen activator (tPA)* gene expression is promoted by the cAMP/PKA signaling pathway in bovine and rat SCs^19^. tPA is a protease that degrades the tight junction proteins to regulate the dynamics of the BTB^20^. Moreover, the PI3K/Akt/mTOR signaling pathway is involved in the development of BTB^21^ (Fig. 1, panel A).

The calcium signaling pathway may also affect the dynamics of BTB in an FSH-dependent manner. By increasing cAMP levels^22^, FSH causes an influx of Ca^2+^ across the cytoplasmic membrane via voltage-gated and voltage-independent calcium channels^23^ (Fig. 1, panel A). Increased intracellular calcium levels activate calmodulin (CaM) and CaM kinases that can influence the cytoskeletal structure of SCs and the phosphorylation of transcription factors, including CREB^24^.

#### Follicle-stimulating hormone regulates the maintenance and differentiation of the spermatogonial pool

SCs create a microenvironment by secreting growth factors, nutrients, and other molecules that are critical for the maintenance and differentiation of the spermatogonial pool. The pivotal role of FSH in these processes has been confirmed by the evidence that altered FSH signaling in immature or mature SCs results in decreased SSC proliferation and maturation^25^. In particular, FSH up-regulates the gene expression of *GDNF* and *FGF2* that promote SSC self-renewal^26^.

FSH can affect SSC proliferation and differentiation also by activating the phospholipase A2 (PLA2) pathway promoting the secretion of prostaglandin E_2_ (PGE_2_) (Fig. 1, panel A). The latter stimulates the self-renewal of SSCs and inhibits their differentiation^27^. Regarding the differentiation process, SCF secretion is regulated by FSH^28^ and, according to another study, enhances SSC differentiation in vitro^29^.

SCs play a crucial role in providing nutritional support to GCs via a mTOR mechanism. Rapamycin (mTOR inhibitor) can alter several metabolic parameters, including glucose consumption and mitochondrial complex III protein levels in human SCs^30^. mTOR dysregulation has also been associated with the onset of metabolic disorders, including obesity and diabetes mellitus (for the review, see ref. ^31^). Furthermore, treatment with glucagon-like peptide-1 (GLP-1) increased mTOR phosphorylation at Ser2448 and reduced mitochondrial membrane potential and oxidative damage in human SCs^32^. These findings suggest that dysregulated mTOR may have a role in subfertility or infertility associated with metabolic disorders.

### The IGF system

IGF1, IGF2, and insulin are the major molecules belonging to the IGF system. They appear to modulate many metabolic and development processes. The expression of the IGF1 receptor (IGF1R) and IGF1 role in the regulation of SC proliferation have been demonstrated^33^.

Interestingly, evidence indicates a possible relationship between the IGF system and FSH. The inhibition of both FSHR and IGF1R causes a decrease in testicular weight, but in SC-*Insr*;*Igf1r* KO mice the reduction was greater than in SC *Fshr* KO^34^, suggesting that FSH-induced proliferation of immature SCs is mediated, at least in part, by IGF1. Consistent with this hypothesis, our more recent studies have shown that FSH-stimulated ERK1/2 and Akt phosphorylation occurs by an IGF1R-dependent mechanism in porcine SCs^35^.

Interestingly, insulin can also exert proliferative effects like IGF1, by binding IGF1R. However, higher insulin concentrations than those of IGF1 are required to produce the same biological response^36^. A previous study has proved that insulin can influence the effects of FSH on porcine SCs, reducing the secretion of AMH and inhibin B from these cells and promoting cell proliferation^37^.

Collectively, these studies demonstrated that the IGF system could play a fundamental role in the endocrine and paracrine networks involved in the regulation of SC proliferation. By interacting with IGF1R and INSR, IGF1, IGF2, and insulin can activate the PI3K/Akt and ERK1/2 pathways (Fig. 1, panel B).

PI3K is a kinase that regulates the activity of several proteins involved in cell metabolism, growth, proliferation, and survival. Activation of IGF1R promotes the phosphorylation of insulin receptor substrate (IRS) 1, which is an adapter protein to facilitate the activation of downstream targets^38^. Consequently, IRS1induces the activation of PI3K^39,40^ which in turn phosphorylates PIP2 PIP3, which is bound by AKT.

Finally, Akt activation triggers many downstream targets involved in regulating metabolic and growth functions (Fig. 1, panel B).

In addition to the PI3K/AKT signaling pathway, the IGF system can activate ERK1/2 independently of PI3K/AKT. More specifically, the activated receptor and IRS proteins trigger both Shc-transforming protein 1 (Shc) and growth factor receptor-bound protein 2 (Grb2), which in turn binds to the son of sevenless (Sos) protein. The latter converts the inactivated form bound to Ras GDP (Ras-GDP) to the activated form bound to Ras GTP (Ras-GTP). Ras-GTP activates downstream effectors, such as Raf, which stimulates Mek^41^. Finally, Mek phosphorylates and activates ERK which, in turn, regulates gene expression by promoting cell proliferation^42^ (Fig. 1, panel B).

#### Insulin resistance may interfere with the follicle-stimulating hormone-induced gene expression

Evidence in recent years has shown the involvement of IGF1R in the FSH signaling pathway.

According to the study by Law and Hunzicker-Dunn, FSH-induced Akt phosphorylation is inhibited in rat granulosa cells treated with a covalent inhibitor of IGF1R (NVP-AEW541)^43^, leading to a decreased expression of genes involved in the germline proliferation. Consistent with this evidence, in the male gonad, the IGF system appears to have an essential role in testicular development, via this interaction with the FSH signaling pathway^34^.

The relationship between these two pathways is linked to the activity of PKA which, as previously mentioned, can phosphorylate Ser or Thr amino acid residues of different proteins (Fig. 1, panel C). One such protein is myosin-phosphatase 1 (MYPT1). Therefore, binding of FSH to its receptor activates AC through Gɑs. AC converts ATP to cAMP, which in turn activates PKA. PKA phosphorylates MYPT1 which results in an activation of PP1 (Fig. 1, panel C). Subsequently, PP1 promotes the dephosphorylation of inhibitory Ser/Thr residues on IRS1, including Ser^789^^43^.

In insulin-resistant conditions and T2DM, increased phosphorylation of IRS-1 is a common finding^44^. Notably, phosphorylation of Ser^307^ can inhibit IRS1 signaling, leading to reduced activation of insulin-induced PI3K and MAPK signaling pathways^45^. Being IRS1 a mediator of the FSH signaling pathway, this could explain the reduced efficacy of FSH treatment found in infertile patients with insulin resistance^46^.

### Androgens

AR is expressed in SCs, Leydig cells, and peritubular myoid cells (PTMCs)^47^. During fetal life, AR is not expressed in rat SCs, and its expression increases progressively after birth^47^, suggesting that AR-induced effects on SCs may have a prevalent role during postnatal development.

Androgens promote gene expression by interacting with their receptor in the cytoplasm^48^. The latter activates Src-promoting EGF receptor (EGFR) stimulation and consequently, CREB phosphorylation^49^.

**Fig. 2:**
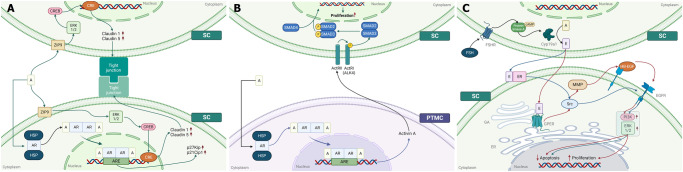
Effect of sexual hormones on Sertoli cell differentiation, proliferation, apoptosis. Androgens directly promote the differentiation of Sertoli cells (**A**). By binding its receptor in the cytoplasm, A promotes the expression of p21Cip1 and p27Kip gens, which induces cell cycle exit. Furthermore, A can interact with the cell membrane receptor ZIP9, which promotes BTB formation by increasing Claudin-1 and -5 gene expression. Androgens indirectly promote the proliferation of Sertoli cells (**B**). Androgens bind their receptor, which in turn promote *activin A* gene expression. Activin A is released in the interstitial space and interacts with ActRII. The latter activates ActRI which phosphorylates SMAD2 and SMAD3. Subsequently, phosphorylated SMAD2-SMAD3 oligomerizes with SMAD4. This oligomer translocates to the nucleus to influence gene transcription. Estrogens promote proliferation and inhibit apoptosis in immature rat Sertoli cells (**C**). E can influence SC proliferation (pathway indicated by blue arrows) by interacting with mERα, which in turn activates Src. Src phosphorylates EGFR to activate PI3K and ERK1/2 signaling pathways that promote SC proliferation. Furthermore, by binding GPER, E inhibits apoptosis of SCs (pathway indicated by red arrows). GPER activates Src, which stimulates MMP activity. MMP releases HB-EGF from the plasma membrane. HB-EGF interacts with its receptor leading to the activation of the PI3K and ERK1/2 signaling pathways and, subsequently, the expression of genes involved in the inhibition of apoptosis. A androgen, ActRI type I activin A receptor, ActRII type II activin A receptor, AR androgen receptor, ARE androgen response element, BTB blood–testis barrier, cAMP cyclic adenosine monophosphate, CRE cAMP response elements, CREB cAMP-response element binding protein, cyp19a1 cytochrome P450 family 19 subfamily A member 1, E estrogen, EGFR epidermal growth factor receptor, ER endothelial reticulum, ERK1/2 extracellular signal‑regulated protein kinase ½, FSH follicle stimulating hormone, FSHR follicle stimulating hormone receptor, GA Golgi’s apparatus, GPER G protein-coupled estrogen receptor 1, HB-EGF heparin-binding epidermal growth factor-like growth factor, HSP heat shock protein, mERα membrane estrogen receptor α, MMP metalloproteinase, PI3K phosphoinositide 3-kinase, PKA cAMP-dependent protein kinase, PTMC peritubular myoid cells, p21cip1 cyclin-dependent kinase inhibitor 1, p27kip1 cyclin-dependent kinase inhibitor 1B, SC Sertoli cell, ZIP9 zinc transporter SLC39A9.

By interacting with the intracellular AR, androgens up-regulate the expression of the cell cycle inhibitors p21Cip1 and p27Kip1, inhibiting SC proliferation in the rat^50^ (Fig. 2, panel A). After the cessation of the proliferative phase, one of the most important processes during SC maturation is the establishment of the BTB, in which androgens appear to play a pivotal role. Indeed, they promote the expression of many factors associated with functional maturation, and other proteins involved with SC tight junctions^51^. Moreover, androgens can regulate the expression of *claudin-1* and −*5*, which are components of tight junctions, by interacting with zinc transporter ZRT- and Irt-like Protein (ZIP) 9^52^ (Fig. 2, panel A).

PTMC secretion can influence SC functions^53^ and AR is expressed in PTMC during fetal and postnatal life. It has been proposed that androgens, by interacting with their receptors, promote *activin A* gene expression which, in turn, acts as a paracrine factor that stimulates SC proliferation. (Fig. 2, panel B). Consistent with this hypothesis, PTMC-*Ar*^−/y^ transgenic mice showed decreased testicular weight and sperm count which could be a consequence of decreased SC number^54^.

### Estrogens

SCs secrete 17ß-estradiol (E_2_) in immature rats in response to FSH which stimulates the expression of *Cyp19a1*^55^, the enzyme that converts T to E_2_. FSH interacts with its receptor and activates the cAMP/PKA signaling pathway leading to the activation of CREB and steroidogenic factor 1, which in turn promote the expression of aromatase^56,57^ (Fig. 2, panel C). Aromatase is not only expressed in SCs but also in Leydig cells, spermatocytes, and spermatids^58,59^. E_2_ production by SCs progressively declines with age^60^ and Leydig cells and/or GCs become the main source of estrogens during adulthood^58,61^.

Estrogens exert their effects by interacting with two types of ER, called ERα (or ESR1) and ERβ (or ESR2), that are respectively involved in the proliferation and differentiation of SCs^62^ (Fig. 2, panel C). Furthermore, estrogens can act through a non-classical signaling pathway by interacting with ER located near or at the cytoplasmic membrane, truncated variants of ERα, or G protein-coupled ER (GPER or GPR30)^63^.

Moreover, E_2_, by binding to ERβ, induces SCF expression in SCs isolated from human fetal testes between 16 and 28 weeks of gestation, promoting SSC proliferation and inhibiting the apoptosis of these cells^64^. Consistent with this, another study showed that E_2_ increased SCF expression in the seminiferous tubules of adult rats. However, the results also demonstrated that a high amount of estrogens inhibit *c-Kit* expression, increase apoptosis, and decrease SSC proliferation^65^.

As for G protein-coupled estrogen receptor 1 (GPER), this receptor has been immunodetected in the endoplasmic reticulum and Golgi apparatus^66^ and its stimulation activates Src which, in turn, promotes the activation ERK1/2 and/or PI3K signaling pathways promoting SC proliferation and survival^67^ (Fig. 2, panel C).

## Obesity influences various molecular signaling pathways in both immature and mature Sertoli cells

The molecular pathways discussed above are compromised in overweight and obesity. They can directly and indirectly alter male fertility through various mechanisms, including hormonal changes, epigenetic modifications, chronic inflammation status, and impaired glucose metabolism.

### Chronic inflammation

Obesity is associated with a chronic inflammatory state that can affect the hypothalamic–pituitary–gonadal (HPG) axis through the production of cytokines. TNF-α and interleukin (IL)-1β could reduce T production by Leydig cells^68^. IL-6, produced in large quantities during inflammation, could induce apoptosis of prepubertal SCs in mice through the Stat3/FoxO1 signaling pathway^69^.

Numerous studies have shown that inflammation, obesity, hyperglycemia, and hyperlipemia phosphorylate the IRS1 protein^70^. Increased phosphorylation of IRS1 has also been reported in insulin-resistant rodents^71,72^. In turn, this causes the dissociation of receptor tyrosine kinase/IRS1 and/or IRS1/PI3K, preventing activation of PI3K^73,74^ or increased degradation of IRS1^75^. Therefore, obese children may have a disruption in the IGF1R-dependent PI3K/AKT/mTORC signaling pathway. This may explain why obese or overweight prepubertal children have been found to have smaller testicular volume than their normal-weight peers of the same age^76^. Insulin-resistant obese children also have lower serum AMH levels than age-matched healthy controls^77^, suggesting fewer number of SCs^15^. Consequently, obese boys have lower AMH levels during the pubertal process compared to healthy controls^78^. In turn, the reduced number of SCs could stimulate fewer SSCs and, therefore, reduce sperm production.

Adipocytokines play a crucial role in the development of obesity-related complications and inflammation. A recent study reported that adult human SCs treated with a supraphysiological concentration of adipocytokines could downregulate the expression of *FSHR*, *bone morphogenetic protein 4* (*BMP4*), *GDNF*, *FGF2*, *leukemia inhibitory factor* (*LIF*), and upregulate *CYP26A1*^79^. Therefore, according to these findings, exposure to obesity-related inflammatory cytokines before the onset of puberty could be compatible with low FSH responsiveness and a reduced capacity of SCs to produce GDNF and stimulate spermatogenesis later in life.

Wagner and collaborators evaluated testicular steroidogenesis in rats with prepubertal onset of obesity, demonstrating that the number of Leydig cells was decreased while the number of testicular macrophages and the concentration of TNF-α were increased^80^. In this study, the effects of a high-fat diet (HFD) on rat SCs were not evaluated. However, the absolute testis weight was decreased in both rats with short- and long-term obesity^80^. Since testis weight in the prepubertal phase is mainly given by the number of SCs, possible damage to SCs and an alteration of their proliferation capacity caused by inflammatory cytokines and macrophages cannot be excluded. This lays the foundations for the hypothesis that exposure to adipocytokines during the prepubertal period can also interfere with SC proliferation, compatible with the development of oligozoospermia in reproductive age.

### Hyperleptinemia

Serum leptin levels are higher in obese patients and in rats fed long-term HFD^81^. By interacting with its receptor (LepR), leptin activates many signaling pathways, including Janus kinase 2/signal transducers and activators of transcription (JAK2/STAT), ERK, and PI3K^82^. Evidence has shown that leptin could restore fertility in leptin-deficient ob/ob mice^83,84^. On the other hand, elevated leptin levels have been associated with azoospermia^85^, abnormal sperm parameters^86^, and fertilization capacity^87^, increased production of reactive oxygen species (ROS), inhibition of steroidogenesis, and impaired testicular maturation^88,89^.

Leptin plays a crucial role in triggering the onset of puberty because it is a key element that links the energy state of the entire organism with male reproductive function^90^. Evidence has shown that leptin can promote the phosphorylation of several members of the IRS factor family in the hypothalamus^91,92^. As previously mentioned, phosphorylated IRS1 is frequently found in insulin resistance, and, more importantly, phosphorylation of IRS inhibitory sites may lead to impaired interplay between FSH and IGF1 in SCs.

Other studies showed that the administration of leptin increased hypothalamic mTOR activity and the administration of the mTOR inhibitor rapamycin decreased the expected effect of leptin^93^. Furthermore, HFD reduced hypothalamic mTOR signaling^94^, suggesting a possible role of mTOR in the development of leptin resistance. Interestingly, mTOR is involved in the control of spermatogenesis, testis physiology, and SC metabolism, and fascinatingly, LepR expression has been identified in human SCs where it modulates their metabolism, regulating acetate production and glycolysis^95^. This appears to be a direct mechanism that explains the association between obesity and male infertility since leptin can interfere with the metabolic support of spermatogenesis by SCs. Moreover, by activating LepR in SCs, leptin can modulate the phosphorylation pattern of IRS1 and mTOR signaling, both of which are crucial in the functioning of prepubertal and adult SCs. Studies are needed to evaluate whether the influence of leptin on SC metabolism is exerted by the PI3K/AKT/mTOR signaling pathway.

Recently, Wang and collaborators have shown that hyperleptinemia can affect the development of BTB in prepubertal rats. In this study, elevated leptin levels reduced the protein expression of ZO-1, claudin 5, and occludin^96^. Furthermore, the expression of *AR* and other steroidogenic genes (*steroidogenic factor 1*, *steroidogenic acute regulatory protein*, and *CYP11a1*) was downregulated^96^, suggesting that obesity-related hyperleptinemia may, directly and indirectly, influence SC maturation in mice.

### Hyperinsulinemia, insulin resistance, and type 2 diabetes mellitus

According to in vitro evidence, insulin modulates prepubertal SC function. In porcine SC cultures, co-incubation with insulin and FSH downregulated the production of AMH, inhibin B—markers of SC function^15^—and *FSHR*^37^. Furthermore, incubation with insulin increased the proliferation of immature SCs^37^, and, therefore, insulin resistance occurring in obese boys may disrupt this process, leading to a depletion of the SC pool. Concordantly, another study showed that median inhibin B and T levels during puberty were significantly lower in overweight or obese boys compared to controls^78^. Moreover, the same study reported that insulin was a negative predictor of AMH levels^78^.

Wang and colleagues showed that the mRNA levels of PI3K, Akt, and Stat3 were significantly downregulated, while the expression of FoxO1, FasL, and IL-6 were significantly higher in SCs from 8-week-old male mice with T2DM^69^. FoxO1 is a downstream target of PI3K/Akt and IL-6/Stat3 signaling pathways^97^ which can induce apoptosis through the expression of downstream apoptotic factor^98^. Under physiological conditions, Akt promotes Ser256 and Thr24 phosphorylation of FoxO1, inhibiting its transcriptional activity and insulin resistance^99^, while IL-6, by binding to its receptor, induces the phosphorylation of Stat3 and promotes the expression of FoxO1^100^. These results suggest that inhibition of the PI3K/Akt pathway or increased IL-6/Stat3 signaling observed in T2DM mice could induce SC apoptosis, leading to abnormal spermatogenetic function.

Interestingly, T2DM-associated SC apoptosis is due to PI3K/Akt-mediated downregulation of testicular vascular endothelial growth factor (VEGF), leading to impaired testicular microcirculation and infertility^101^.

### Hypotestosteronemia and relative hyperestrogenism

The effects of estrogens on testicular function seem to depend on their concentration. In vitro evidence has shown that the physiological concentration of estrogens in the testis promoted GC survival^102^, while increased amounts, such as those observed in obesity, induced GC death by apoptosis^65,103^, Similarly, a low concentration of E_2_ increased the number of spermatids, whereas a high concentration altered testicular morphology^104^. Consequently, idiopathic infertile patients with increased E_2_ concentrations in the testis and reproductive fluids have been reported^105^.

Elevated estrogen levels can induce apoptosis through intrinsic and extrinsic apoptotic pathways. Estrogens upregulate the expression of Fas and FasL^65,102^ and modulate the expression of Bax in testicular cells^65,106^. Treatment with estradiol benzoate increased the rate of apoptosis in rat testes by decreasing the enzymatic activity of superoxide dismutase and catalase, leading to increased lipid peroxidation and reduced antioxidant defense^106^. Furthermore, it was reported that pachytene spermatocytes isolated from rat testes and treated with E_2_ showed reduced mRNA levels of cyclin-A1 and cyclin-B1 and increased expression Bax, through the activation of the Egfr/ERK/c-Jun pathway^107^. These findings suggest that E_2_ may affect rat GC apoptosis without the mediation by SCs.

Another mechanism through which E_2_ could promote GC apoptosis is the downregulation of *c-kit* expression, which is essential for GC survival^65^.

In vitro studies have shown that E_2_ regulates the glucose metabolism of SCs. In detail, estrogens appear to modulate glucose transporter type 3 (Glut3) and Ldh mRNAs^108^. One study demonstrated that exogenous estradiol benzoate treatment in 4-week-old (prepubertal) rats caused subsequently azoospermia^109^. Furthermore, Glut3 mRNA was upregulated, while monocarboxylate transporter 2 (Mct2) and monocarboxylate transporter 4 (Mct4) mRNAs were downregulated^109^, suggesting that estrogens may promote the internalization of glucose but inhibit the export of lactate which is essential to support GCs. Another study showed that both E_2_ and bisphenol A increased oxidative stress and decreased insulin, Ir, Irs1, PI3K, and Glut2 protein levels in 3-month-old rat testis^110^. Moreover, decreased levels of 3-β-hydroxysteroid dehydrogenase (3β-HSD), 17-β-hydroxysteroid dehydrogenase (17β-HSD), steroidogenic acute regulatory protein, and T were also observed^110^. Overall, these findings indicate that increased amounts of free serum estrogens before the onset of puberty may negatively impact SC metabolism, interfering with the ability of these cells to feed GCs and also directly induce GC apoptosis, thus causing azoospermia.

Regarding T, Rato and colleagues demonstrated that T deficiency promoted glycogenesis and impaired glucose metabolism in mature rat SCs^111^, which may lead to abnormal nutritional support for GCs. Importantly, a 2015 study showed that 21-day-old mice fed an HFD had abnormal tight junction biomarker protein content and decreased levels of T and androgen receptors^112^, suggesting that obesity may affect BTB development through the downregulation of the androgen signaling pathway.

Inhibin B and T levels during puberty are significantly lower in overweight or obese boys than in normal-weight boys starting at age 12 for inhibin B and from age 14 onwards for T^78^. Androgens could enhance the expression of *GDNF*, *SCF*, and *FSHR* genes in SCs^113,114^. Furthermore, T could promote the expression of *c-kit* in rat spermatogonia, improving the responsiveness of these cells to SCF^115^. Azarniad and colleagues demonstrated that experimental diabetes mellitus in rats could reduce the expression of *Gdnf, Gfrα1, c-ret*, and *Bcl-6b*, which in turn leads to significant suppression of the self-renewal process of SSCs^116^. Interestingly, rats showed low T levels, suggesting that hypotestosteronemia and diabetes mellitus might interfere with GC proliferation through disruption of the GDNF signaling pathway^116^.

Therefore, prepubertal obesity may lead to impaired spermatogenesis later in life due to altered BTB integrity, caused by obesity-related abnormal levels of sex hormones.

## Interplay between glucose metabolism and Sertoli cells

Glucose intake and SC metabolism are essential for GC growth and development. The PI3K signaling pathway plays a crucial role in regulating these processes via a signaling pathway involving mTOR and AKT. The mTOR signaling pathway intervenes in the conversion of amino acids into proteins, while, importantly, the AKT signaling pathway regulates the expression of GLUTs and phosphofructokinase enzymes in immature SCs^117,118^ to support their metabolism and proliferation.

In mature SCs, once the glucose is introduced, it is converted into metabolic substrates necessary for GC development, such as lactate^119^. The latter is exported from SCs to GCs specifically through MCT4^120^. All these processes are influenced by insulin and sex hormones. Evidence demonstrated that SCs could modulate the expression of *GLUT1* and *GLUT3* under insulin deprivation conditions to ensure adequate amounts of lactate for GC development^121^. Furthermore, in the same study, it was observed that lack of insulin downregulates the expression of *LDH A*, which in turn promotes the conversion of pyruvate to lactate and *MCT4*^121,122^. These results were concordant with another study reporting that insulin and FSH increased lactate production by immature SCs^123^, suggesting a positive correlation between insulin level and lactate. Regarding sex hormones, Martins and colleagues proved that 5α-dihydrotestosterone (DHT) and E_2_ regulate the expression of Glut1, Glut3, and Pfk1 transcripts^108^. However, only DHT downregulated Glut1 protein levels and increased Ldh activity in SCs extracted from 20-day-old rats (prepubertal)^108^. Accordingly, Rato and colleagues found that DHT modulates glucose consumption and lactate excretion by rat SCs by downregulating the expression of *Ldha* and *Mct4*^124^.

Overall, this evidence indicates that obesity-related insulin resistance and abnormal levels of sex hormones can affect the metabolism of both immature and mature SCs, thus interfering with their function in both prepubertal and post-pubertal life.

## Influence of gut hormones on Sertoli and germ cell physiology

Gut hormones are involved in the modulation of the sense of satiety, appetite, lipid, and carbohydrate metabolism, and also have effects at a hemodynamic level.

Their serum concentrations are altered in obesity. Ghrelin levels are generally low in obese patients compared to normal-weight subjects^125^. Similarly, GLP-1 secretion is impaired in patients with obesity or T2DM^126,127^. Evidence has shown that HFD is associated with increased glucose-dependent insulinotropic polypeptide (GIP) levels^128^, which in turn leads to higher fat distribution^129^. Similarly, patients who are obese or have T2DM show high secretion of GIP^130,131^. Cholecystokinin (CCK) levels appear to be reduced in the case of obesity^132^, although contradictory data have been reported in this matter^133^. Furthermore, obesity alters the pattern of secretin release, reducing the peak secretion after the oral glucose tolerance test^134^. Overall, these findings have led researchers to hypothesize that the impaired secretion of gut hormones may contribute to the onset of appetite disorders, reduced lipolysis, and hemodynamic alterations that occur in patients with obesity.

Understanding the impacts that these hormones have on the physiology of SCs could allow us to establish a link between infertility and the dysfunction of these cells. Human SCs express obesity-related genes (*ORG*, *MC4R*, *GNPDA2*, *TMEM18*, and *FTO*), and the expression of GNPDA2 and TMEM18 proteins in SCs has been reported to increase after incubation with leptin and ghrelin, leading to a modulation of the nutritional support provided for spermatogenesis^135^. Ghrelin has been suggested as a fertility-related energy sensor, capable of influencing SC metabolism dependent on energy uptake. Its receptors are expressed in mature human SCs, where binding to ghrelin modulates glucose uptake via GLUT expression, lactate production via LDH activity, and mitochondrial function. Incubation of mature human SCs with ghrelin at a concentration mimicking that of obese patients resulted in downregulation of ghrelin receptor and GLUT1, higher pyruvate consumption, and acetate production, compared to those incubated with a concentration simulating that of normal weight^136^. A reduction in SC metabolism at this stage may result in reduced proliferation, impacting future spermatogenetic potential.

Another gut hormone that appears relevant to SC physiology is GLP-1. Its receptors are expressed in human SCs^32^. Exposure to GLP-1 has been shown to decrease glucose consumption and increase lactate production at concentrations of 0.01 and 1 nM that mimics its post-prandial GLP-1 levels, and increase mTOR phosphorylation, decrease mitochondrial membrane potential and oxidative damage at 100 nM (a concentration that mimics pharmacological levels)^32^. Therefore, GLP-1 appears to support SC metabolism and lactate production, which is important for supporting spermatogenesis. The impact of GLP-1 on immature SCs has not been investigated. A Danish population-based study on thousands of children and adolescents recently provided evidence of elevated serum GLP-1 levels under fasting conditions in the obese group, compared to peer controls, and these levels were related to cardiovascular risk factors, such as insulin resistance, hypertension, hyperglycemia, and dyslipidemia^137^. Understanding the effects of these abnormal levels on immature SC function represents an interesting field of study, which may provide further evidence for a link between childhood obesity, SC dysfunction, and thus, future impairment of spermatogenesis.

Regarding other gut hormones, data on the expression of receptors for GIP, CCK, and secretin in SCs, as well as functional studies aimed at understanding their impact on SC physiology, are lacking.

The role that gut hormones have on GCs or Leydig cells should be emphasized as this evidence is also useful for understanding the pathogenesis of infertility associated with obesity.

Ghrelin appears to play a protective role in the testis against heat stress, to which, for example, patients with varicocele are exposed. This is supported by an animal study, where the ghrelin injection was able to reverse heat stress-induced testicular damage in rats^138^. Therefore, low circulating levels of ghrelin, as seen in obese adolescents and adults, may favor SC and spermatogenesis injury in case of heat stress exposure.

GLP-1R is expressed in Leydig cells where it appears to influence steroidogenesis^139^. This receptor has also been identified in human spermatozoa, where incubation with a GLP-1 agonist has been shown to trigger the PI3K/AKT signaling pathway through PKA, LDH, and G6PDH activity^140^. Accordingly, treatment with a GLP-1R agonist produced an improvement in sperm parameters in obese adult patients, although an effect due to weight loss on the observed results cannot be excluded^141^. There is no evidence available on the effects of these drugs on testicular function in childhood or adolescent obesity.

GIP is believed to be a means by which food intake exerts its influence on fertility. It is present in seminal plasma and in higher concentrations in obese men compared to normal weight controls^142^. Its receptor (GIPR) has been identified in mouse spermatids, where it has been shown to play a crucial role in sperm–egg function^143^. Furthermore, GIP concentrations in the testes, as well as Gipr expression, were affected by overeating or starvation, supporting the GIP signaling pathway as a target that regulates fertility potential depending on energy supply.

The CCK type 1 receptor has been found in boar sperm, where incubation with CCK, in the presence of bicarbonate, has been shown to positively influence sperm motility and capacitation^144^, likely through the adenylyl cyclase/cAMP/protein kinase A signaling pathway^145^. Therefore, altered levels of CCK, such as those found in obese patients may negatively affect sperm function.

Although secretin and its receptor are expressed in GCs, especially in round spermatids^146^, their influence on GC physiology is unknown.

In conclusion, current evidence supports the influence of ghrelin and GLP-1 on mature SC metabolism, in turn regulating the energy supply for spermatogenesis. Ghrelin, GLP-1, GIP, and CCK also modulate sperm and Leydig cell function, providing further evidence for a link between obesity and infertility.

## Discussion

**Fig. 3:**
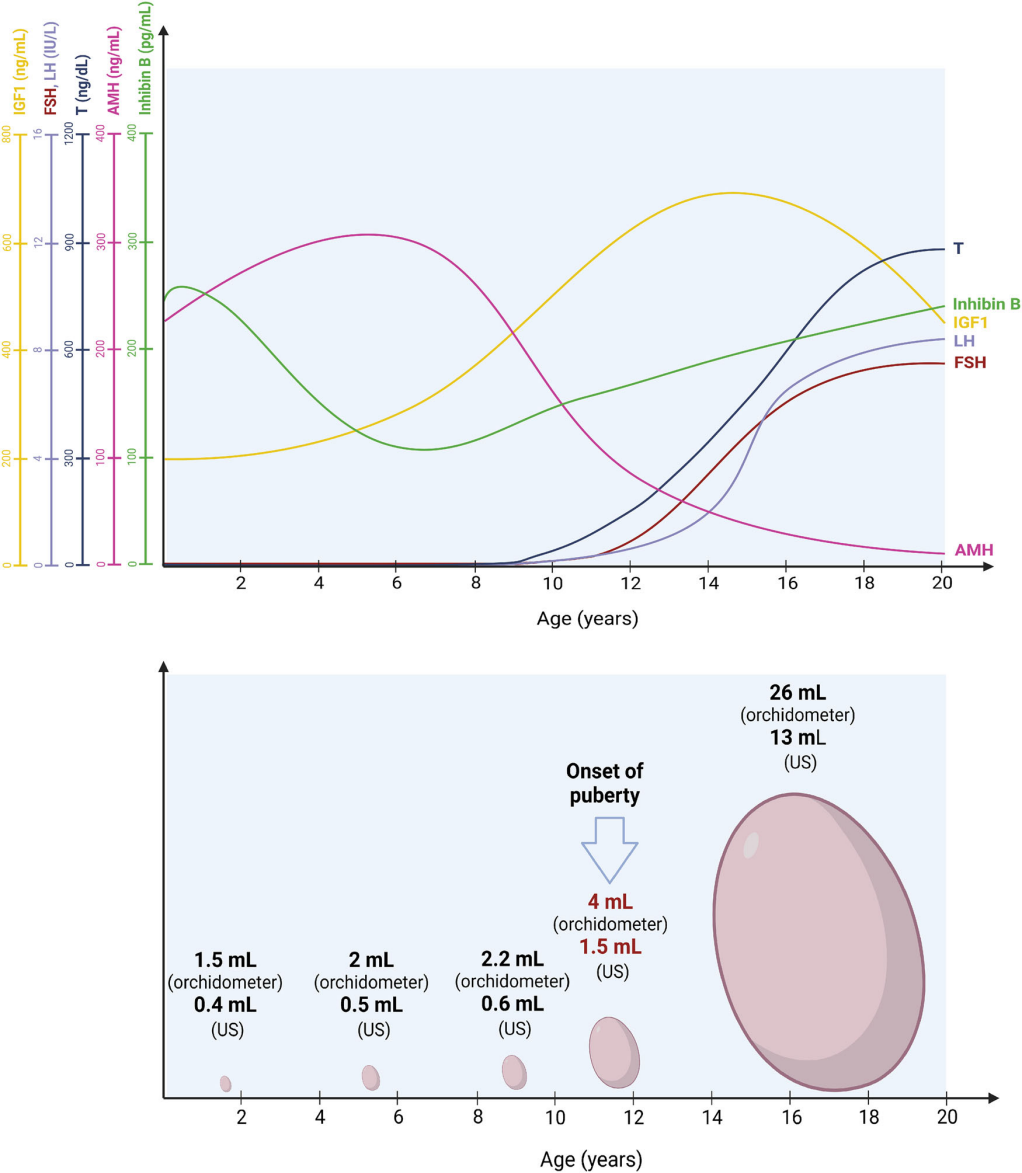
Testicular growth over time. The upper panel shows the variation in hormone serum levels from birth to the age of 20. The hormonal peaks that occur during mini-puberty are not represented. The sources used for the reference values are Juul and Skakkebæk^152^ for IGF1; Soldin et al.^153^ for FSH and LH; Holmes et al.^154^ for total testosterone [Edelsztein et al.^147^ for AMH; and Kelsey et al.^155^ for Inhibin B. The bottom panel shows the increase in testicular volume over time. Values were plotted using the 50^th^ percentile of orchidometric and ultrasound data published by Joustra et al.^156^. AMH anti-Müllerian hormone, FSH follicle-stimulating hormone, IGF1 insulin-like growth factor 1, LH luteinizing hormone, US ultrasonography, T testosterone.

The association between adult obesity and infertility has been studied by several researchers^4,5^. More intriguing but less studied, however, are the consequences of childhood and adolescent obesity on future fertility. Although the testis has always been considered a silent gland until the activation of the HPG axis during puberty, it is the site of intense metabolic activity during the pediatric age^15,147^. SCs proliferate intensely until puberty, remaining in a state of immaturity, during which they secrete large quantities of AMH. As puberty begins, they mature, lose the ability to proliferate, begin to produce less AMH, and, importantly, gain the ability to stimulate the proliferation and differentiation of SSCs to support spermatogenesis (for review see refs. ^15,147^). However, each SC is capable of supporting the differentiation of only a finite number of SSCs^148^ and the number of spermatozoa produced by the testis once maturity is reached depends on the final number of SCs^149^ Consequently, all processes capable of negatively influencing SC proliferation in pediatric age can compromise their proliferation and/or maturation, leading to a lower number of SCs, which, consequently, will be able to stimulate a small number of SSCs, favoring a condition of irreversible oligozoospermia, since the SCs can no longer proliferate (Fig. 3).

Evidence has shown a negative impact of metabolic diseases arising during childhood or puberty on testicular function later in life in terms of lower testicular volume and impaired sperm output^150^. Other data supporting the presence of testicular dysfunction in obese children are the lower testicular volume^76^ and serum AMH levels^77,78^ reported in overweight and obese boys compared to their healthy peers. Both these data suggest a poor expansion of the Sertolian pool at the testicular level. Interestingly, the longer time to onset of puberty onset, which has been reported in obese children^76^, has recently been associated with worse sperm output in adulthood^151^, potentially leading to speculation about the existence of a poorer sperm output in men who had obesity in childhood and later onset of puberty.

Here, we have provided a comprehensive overview of the mechanisms that may explain the association between childhood obesity and impaired spermatogenesis later in life. Direct evidence indicates that adipocytokines downregulate the expression of *FSHR* and *GDNF* in immature SCs. Indirect data suggest that they may also decrease SC proliferation as their elevated levels have been found to reduce testicular weight in prepubertal rats. Obesity-related hyperleptinemia alters BTB formation as well as the metabolism of adult SCs. Furthermore, insulin triggers the proliferation of immature SCs and the expression of GLUT1, GLUT3, LDHA, and MCT4. All these processes could be altered in case of insulin resistance. Receptor tyrosine kinases, stimulated by IGFs, are involved in the proliferation process of SC alone—before puberty—and together with FSH during the onset of puberty. These receptors are associated with IRS1 and their function changes during metabolic diseases such as insulin resistance. This supports the presence of altered SC proliferation in the case of insulin resistance. Apoptosis of immature SCs occurs in T2DM through downregulating the PI3K/AKT-dependent VEGF pathway and consequent disruption of testicular microcirculation.

**Fig. 4:**
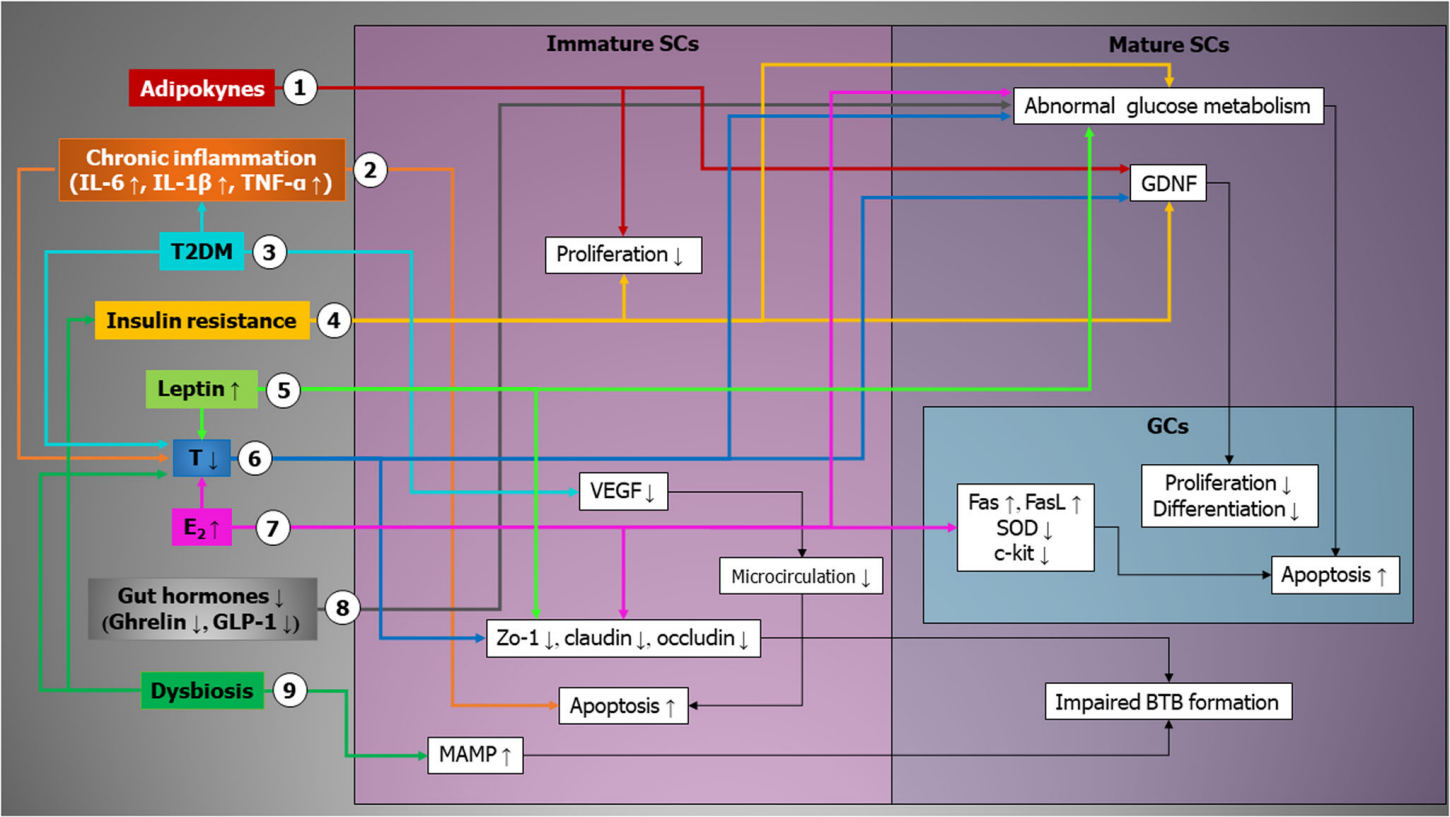
Summary of the mechanisms through which obesity can alter Sertoli cell physiology. (1) Adipokines affect the proliferation of SCs and GCs through the downregulation of FSHR and GDNF in immature and mature SCs, respectively. (2) Inflammatory cytokines (TNF-α and IL-1β) can reduce the T production by LCs. Increased IL-6 induces SC apoptosis via STAT3/FoxO1 signaling pathway. (3) T2DM is associated with increased IL-6 levels, lower T production, and lower VEGF, which in turn can affect testicular microcirculation leading to immature SC apoptosis. (4) Insulin resistance affects proliferation in immature SCs, glucose metabolism, and the production of extrinsic factors such as GDNF by mature SCs. (5) Hyperleptinemia impairs glucose metabolism in SCs and BTB development reducing the expression of the components of tight junction, *AR*, and other steroidogenic genes. (6) Low T level decreases the expression of *Zo-1*, *claudin*, and *occluding*, which are essential for BTB establishment; reduces the level of GDNF and alters the glucose metabolism in SCs. 7) High E_2_ negatively affects BTB integrity directly and indirectly, decreases T production, impairs glucose metabolism, increases GC apoptosis increasing the expression of *Fas* and *FasL* and reducing *c-kit* expression, and reduces SOD leading to increased lipid peroxidation and reduced antioxidant defense. (8) Low Ghrelin and GLP-1 are associated with abnormal SC metabolism. (9) Dysbiosis is associated with lower T levels, decreased insulin sensitivity, and disruption of BTB integrity^157,158^. More details are given in the text. AR androgen receptor, BTB blood–testis barrier, E_2_ 17-β estradiol, FoxO forkhead box O, Fas Fas cell surface death receptor, FasL Fas ligand, FSHR follicle-stimulating hormone receptor, GC germinal cell, GDNF glial-derived neurotrophic factor, GLP-1 glucagon-like peptide-1, IL interleukin, LC Leydig cell, SC Sertoli cell, SOD superoxide dismutase, T testosterone, TNF-α tumor necrosis factor-α, T2DM type 2 diabetes mellitus, VEGF vascular endothelial growth factor.

Hyperestrogenism that occurs in obese adolescents due to both amplified aromatization and low SHBG (which increases free estrogens), impairs SC metabolism, leading to increased glucose uptake but reduced lactate export, which is of pivotal importance for GC physiology. This is responsible for GC apoptosis, which is also supported by other estrogen-dependent direct mechanisms. Similarly, low T levels alter BTB integrity and SC metabolism, downregulating GLUT1, PFK1, LDHA, and MCT4, resulting in reduced glucose exchange and lactate efflux. This leads to impaired GDNF signaling pathways and consequent suppression of GC proliferation. Furthermore, altered serum levels of ghrelin and GLP1 in obese patients can profoundly influence SC metabolism, downregulating GLUT1, LDH, and mitochondrial function, thus interfering with the production of the energy supply for GCs (Fig. 4). All of these mechanisms may simultaneously be occurring in obese boys, interfering with the delicate metabolic processes that take place in their testes and drawing an intricate picture that could explain why obesity arising in childhood or adolescence can alter the spermatogenetic niche and cause infertility.

All this explains why it is urgent and necessary to study the impact of pediatric obesity on future fertility. Existing evidence to date does not yet allow us to unequivocally confirm this association.

The biggest challenge in this chapter concerns the lack of long-term longitudinal evidence evaluating testicular growth, timing of puberty onset, and sperm output in obese children. In fact, to date, there are no longitudinal studies that have measured sperm parameters in men affected by obesity during childhood. Equally important is to understand the effects of weight loss on obesity-related targets mentioned above (adipocytokines, insulin resistance, hyperestrogenism, low T, gut hormones) and on testicular function in obese boys, to understand whether the weight loss is effective in reversing SC dysfunction and through which mechanisms.
